# Reduction of freezing of gait in Parkinson's disease by repetitive robot-assisted treadmill training: a pilot study

**DOI:** 10.1186/1743-0003-7-51

**Published:** 2010-10-14

**Authors:** Albert C Lo, Victoria C Chang, Milena A Gianfrancesco, Joseph H Friedman, Tara S Patterson, Douglas F Benedicto

**Affiliations:** 1 VA RR&D Center of Excellence-Center for Restorative and Regenerative Medicine, Providence VA Medical Center, 830 Chalkstone Ave, Providence, RI, 02908, USA; 2 Department of Neurology, Warren Alpert School of Medicine, Brown University, Providence, RI, 02912, USA; 3 Departments of Community Health and Engineering, Brown University, Providence, RI, 02912, USA; 4 Butler Hospital, 345 Blackstone Blvd, Providence, RI, 02906, USA

## Abstract

**Background:**

Parkinson's disease is a chronic, neurodegenerative disease characterized by gait abnormalities. Freezing of gait (FOG), an episodic inability to generate effective stepping, is reported as one of the most disabling and distressing parkinsonian symptoms. While there are no specific therapies to treat FOG, some external physical cues may alleviate these types of motor disruptions. The purpose of this study was to examine the potential effect of continuous physical cueing using robot-assisted sensorimotor gait training on reducing FOG episodes and improving gait.

**Methods:**

Four individuals with Parkinson's disease and FOG symptoms received ten 30-minute sessions of robot-assisted gait training (Lokomat) to facilitate repetitive, rhythmic, and alternating bilateral lower extremity movements. Outcomes included the FOG-Questionnaire, a clinician-rated video FOG score, spatiotemporal measures of gait, and the Parkinson's Disease Questionnaire-39 quality of life measure.

**Results:**

All participants showed a reduction in FOG both by self-report and clinician-rated scoring upon completion of training. Improvements were also observed in gait velocity, stride length, rhythmicity, and coordination.

**Conclusions:**

This pilot study suggests that robot-assisted gait training may be a feasible and effective method of reducing FOG and improving gait. Videotaped scoring of FOG has the potential advantage of providing additional data to complement FOG self-report.

## Background

Freezing of gait (FOG) is a common yet poorly understood gait phenomenon in persons with Parkinson's disease (PD). Defined as an episodic inability to generate effective stepping [1], FOG is reported to be one of the most disabling, the second most distressing, and the third most intense parkinsonian symptom [2,3]. Patients often describe FOG as a feeling that their feet are "stuck to the floor" despite attempts to force themselves to walk. Cross-sectional studies indicate increasing prevalence of FOG with duration of disease. Approximately 30% of PD patients experience FOG within 5 years, and nearly 60% after 10 years [4-6]. Predisposing factors that may contribute to FOG include abnormalities of gait such as arrhythmicity and asymmetry [7].

Available pharmacological agents have a limited effect on FOG or other gait symptoms; however, intermittent somatosensory cues, such as simple visual and tactile cues, may alleviate freezing by acting as positive mediators of gait. Nieuwboer and colleagues investigated the potential therapeutic role of external physical cues for individuals with PD who experience FOG (PD+FOG) to improve gait-related mobility in the RESCUE trial [8]. However, simple external cues may not be sufficient to reduce FOG. For example, adding treadmill training to visual and auditory cues was more beneficial than cueing alone in individuals with PD+FOG [9]. The Lokomat (Hocoma, Zurich, Switzerland) is an external device explicitly designed to physically guide repetitive, rhythmic, bilateral lower extremity movements in order to generate a more normal gait cycle. This type of intense stereotyped somatosensory cueing and stimulation may reinforce gait automaticity, thus reducing FOG. The objective of this pilot study was to examine the extent to which FOG and gait arrhythmicity would be ameliorated by using robot-assisted gait training in a small case series. We hypothesized that robot-assisted gait training would reduce FOG frequency and severity, and improve gait. To our knowledge, robot-assisted gait training has not previously been evaluated as a therapy to specifically treat FOG.

## Methods

### Participants

Five individuals with idiopathic PD and primarily "OFF" freezing were recruited from a local Movement Disorders Clinic. Participants were screened at a baseline visit, which included a physical and neurological exam as well as the Unified Parkinson's Disease Rating Scale (UPDRS) assessment. Inclusion criteria were: (1) diagnosis of idiopathic PD by UK Brain Bank criteria, without other significant neurological problems; (2) between the ages of 18-85 years; (3) history of FOG during the "ON" phase of medication by self-report and verified by a neurologist (at screening and baseline); and (4) able to walk 25 feet unassisted.

Exclusion criteria were: (1) unable to understand instructions required by the study (Informed Consent Test of Comprehension); (2) primarily wheelchair bound; (3) presence of medical or neurological infirmity that might contribute to significant gait dysfunction; (4) uncontrolled hypertension > 190/110 mmHg; (5) history of uncontrolled diabetes; (6) significant symptoms of orthostasis when standing up; (7) circulatory problems, history of vascular claudication or pitting edema; (8) body weight over 100 kg; (9) lower extremity injuries or joint problems (hip or leg) that limit range of motion or function, or cause pain with movement; (10) pressure sores with any skin breakdown in areas in contact with the body harness or Lokomat apparatus; (11) chronic and ongoing alcohol or drug abuse, active depression, anxiety or psychosis that might interfere with use of the equipment or testing; (12) inability to participate in and complete the training sessions; (13) diagnosis of atypical parkinsonian syndrome; or (14) implantation of deep brain stimulation.

The Providence Veterans Affairs Medical Center (PVAMC) Institutional Review Board approved the protocol, and informed consent was obtained for all participants. The study was registered on ClinicalTrials.gov (Identifier #NCT00819949).

### Intervention

**Figure 1:**
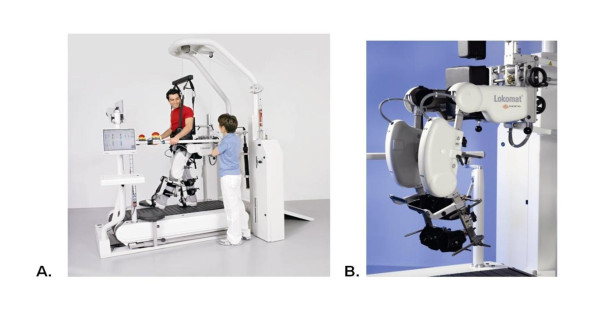
**(A). The Lokomat, an automated gait orthosis on a treadmill with a body weight-support system; (B). Lokomat leg orthosis**.

The Lokomat is a commercially available system that offers mechanical guidance of lower extremity trajectories (Figure 1). The hip and knee components of the exoskeleton are driven by linear back-drivable actuators that repetitively facilitate bilateral symmetrical gait patterns [10,11]. The Lokomat unit is secured to the lower extremity and pelvis using adjustable pads, cuffs and Velcro straps. The system uses a dynamic body weight-support system to support the participant above a motorized treadmill synchronized with the Lokomat.

Participants received 10 sessions of robot-assisted body weight-supported treadmill training (BWSTT) on the Lokomat. Training occurred approximately twice a week for five weeks, and each training session on the Lokomat lasted 30 minutes. All sessions were supervised by a trained research therapist. All participants started with 40% body weight-support and an initial treadmill speed of 1.5 km/h. Body weight-support was used primarily to facilitate an increase in walking velocity; therefore, progression of training across subsequent sessions was standardized by preferentially increasing speed and then unloading body weight-support. Speed was increased to a range of 2.2 to 2.5 km/h before body weight-support was decreased. There was an active attempt to progress the training at each session. By the tenth training session, all participants were walking without body weight-support.

### Outcome Assessments

All outcome assessments were conducted approximately 1 hour after participants took their usual medication to ensure they were in an "ON" phase. Participants were instructed to come to the research facility at the same time and on the same days each week to ensure testing consistency. All outcome assessments were collected at baseline (approximately one week before the first training session) and endpoint (approximately one week after the last training session), and included:

- The Freezing of Gait-Questionnaire (FOG-Q): This self-reported assessment has been shown to reliably detect the impact of FOG and assess the effectiveness of treatment [12,13]. Questions 1-2 pertain to general gait difficulties, while question 3 refers to FOG frequency and questions 4-6 refer to FOG severity. The questionnaire was administered at baseline, before each training session, and at endpoint. In order to reduce recall bias, the baseline FOG-Q score reflects the second time the questionnaire was presented (i.e., prior to Lokomat training at Session 1).

- FOG and Falls Diary: Participants were asked to record the date and number of any FOG episode or fall that occurred throughout the training period. Participants were given the calendar at baseline and it was collected and reviewed at each training session. A fall was defined as an event resulting in a person coming to rest inadvertently on the ground or a level lower than waist height, and not as a consequence of a violent blow, sudden loss of consciousness, or paralysis [14].

- Posture and Gait Score: This score includes questions 13-15 and 29-30 of the UPDRS, and has been used as an outcome measure to assess gait and balance in individuals with PD [9,15].

- Gait Parameters: Spatiotemporal gait characteristics were recorded using a 29-foot instrumented walkway (GAITRite Mat, CIR Systems) calibrated for 25 feet of data collection, placed in a hallway with minimal distractions. Participants completed two walking trials at a comfortable pace down the walkway.

- Gait Rhythmicity, Asymmetry, and Coordination (CV, GA, PCI): These measurements are used to describe bilateral gait coordination, rhythmicity and asymmetry. Coefficient of variation (CV) of spatiotemporal gait parameters is used to describe gait variability, with higher values indicating a more variable gait. Gait asymmetry (GA) is the natural log of the ratio of the swing time of each lower limb, where higher values indicate more asymmetrical gait patterns. Phase coordination index (PCI) assesses the relationship between step time and stride time as well as the variability of that relationship; higher values indicate decreased coordination of the lower extremities [7].

- Parkinson's Disease Questionnaire-39 (PDQ-39): This questionnaire examines 8 dimensions of quality of life specific to PD patients and is scored on a 5-point scale. As a disease-specific questionnaire, the PDQ-39 is highly reliable and valid [16].

- Visual FOG (vFOG): Using a clinican-based scoring method adapated from Schaafsma and colleagues [17], we assessed the frequency and severity of an individual's FOG episodes. A high definition camcorder mounted on a stationary tripod was used. It faced the participant at one end of the 10-meter FOG testing pathway, approximately 5 feet away from where the turns occurred. All participants completed a series of five videotaped walking trials and were asked to stand from a seated position, walk 10 meters, turn, and walk back. Participants completed all five trials continuously, but were allowed to rest between trials if fatigued. The walking trials were completed at baseline, twice each training session (once prior to and once immediately after), and endpoint. The videotapes were coded and scored by a trained neurologist blinded to time point of assessment. The rater was allowed to stop and replay the video during scoring. In order to eliminate a potential novelty or training effect, the trials conducted prior to training at session 1 were used as baseline measurements for data analysis.

### Data Analysis

Self-reported freezing and falls data were each averaged to obtain the number of freezes per day, as well as the number of falls per week throughout the course of the training period. The gait parameters were calculated by GAITRite software (v3.9), and included overall velocity and cadence, as well as limb-specific step length, stride length, and percentage of time spent in swing and double support phases. Limb-specific gait parameters were averaged to obtain a single value; the values of the two trials were then averaged. The CV (standard deviation/mean × 100) was calculated for step length, stride length, stride time and swing time for each participant. GA was calculated as: GA = 100 × |ln (SSWT/LSWT)|, where SSWT and LSWT represent short mean swing time and long mean swing time, respectively [18]. PCI was calculated according to Plotnik and colleagues [18].

The PDQ-39 subsection and standard index (SI) score effect sizes (mean difference/standard deviation at baseline) were calculated according to instructions provided in the PDQ-39 handbook, and compared to reported values of significant meaningful change [19].

In order to generate the vFOG scores, videotapes of the 5 walking trials for each participant at each session were randomized and scored by a trained neurologist, blinded to time point. Frequency of FOG was scored by calculating the mean number of FOG episodes that occurred during the five walking trials under the contexts of: 1) initiation from standstill, 2) open runway walking, 3) onset of turn, 4) turning 180°, and 5) initiation after turning. A "freeze" was defined as an event when the foot appeared to be "stuck," and a visible attempt was made to move, but the foot was unable to proceed as during start hesitations or transient blocks in the middle of motions [17,20]. Severity of FOG was measured by the duration (in seconds) of each freeze that occurred in each of the five contexts previously described. The severity of FOG score was obtained by calculating the mean number of seconds that each FOG episode lasted within each context over the 5 trials, for each videotaped session. Data is reflected as median and interquartile range [25^th ^percentile, 75^th ^percentile] unless otherwise stated.

## Results

Four participants completed all 10 sessions; one participant withdrew after four training sessions due to transportation issues. There were no serious adverse events related to the study. The median age was 62.0 [53.8, 71.5] years, and disease duration was 5.2 [2.7, 8.8] years. The median UPDRS III score was 20.5 [16.8, 24.5]. Participant demographics are presented in Table 1.

**Table 1:** Demographics

	Participant 1	Participant 2	Participant 3	Participant 4
Age (years)	67	57	85	44
Sex	M	M	M	F
Race	White	White	White	White
Height (cm)	170.2	177.8	173.0	175.3
Weight (kg)	59.5	100.0	72.6	66.0
Disease duration (years)	14.0	3.5	0.2	7.0
UPDRS III (ON)	22	10	19	32

### Motor and Quality of Life Outcomes

All participants displayed a reduction of FOG by self-report in response to the intervention. Participants showed a 20.7% reduction in average frequency of freezes per day as recorded on the FOG calendars, with three participants reporting 2-3 fewer episodes of freezing per day. One participant did not report any change in freezes per day, but did report 4 fewer falls per week. There was a 13.8% improvement on the FOG-Q from baseline to end of training (Table 2); specifically, severity of freezing improved 41.7% in "overall" and "initiation" FOG, which correspond to questions 4 and 5 of the FOG-Q.

**Table 2:** Changes in Motor Outcomes Following Robot-Assisted Gait Training

	Participant 1		Participant 2		Participant 3		Participant 4		Median % Change
	*Baseline*	*Endpoint*	*Baseline*	*Endpoint*	*Baseline*	*Endpoint*	*Baseline*	*Endpoint*	
*Freezing of Gait*									
FOG-Q (total)	15.0	13.0	14.0	13.0	15.0	12.0	14.0	12.0	-13.8%
Question 3	3.0	3.0	3.0	3.0	4.0	4.0	3.0	3.0	0%
Questions 4-6	8.0	5.0	6.0	6.0	7.0	4.0	6.0	3.0	-35.4
Average Freezes/Day	8.6	6.4	4.6	1.8	17.4	14.6	3.8	3.8	-20.7%
*Balance*									
Posture & Gait Score	8.0	5.5	6.0	6.0	5.0	5.0	12.0	10.0	-8.3%
*Falls*									
Avg. Falls/Week	0.0	0.0	4.0	2.0	0.0	0.0	12.0	8.0	-16.7%
*Gait*									
Velocity (cm/sec)	106.8	111.3	55.8	72.2	91.6	109.0	58.8	87.7	24.1%
Cadence (steps/min)	114.0	111.4	89.2	88.2	113.9	114.7	112.8	98.4	-1.7%
Stride Length (cm)	112.9	120.2	75.4	97.9	96.5	113.5	62.3	107.5	23.8%
Double Support (%)	26.6	26.4	38.4	32.0	29.6	25.5	41.6	25.4	-15.2%
Swing (%)	36.8	36.9	30.8	34.0	35.0	37.2	29.1	37.2	8.6%
Step Length (cm)	56.2	60.0	37.5	49.1	60.0	57.0	29.8	53.4	18.8%

Gait velocity and stride length improved 24.1% and 23.8%, respectively (Table 2). Participants also demonstrated a reduction in step length CV, swing time CV, and stride time CV, as well as PCI (Table 3). Stride length CV was reduced for three of the four participants. Only one participant demonstrated a decrease in GA.

**Table 3:** Gait Rhythmicity, Symmetry and Coordination

	*Baseline*	*Endpoint*
Swing Time CV (%)	10.2 [9.0, 12.6]	6.7 [6.1, 7.4]
Stride Time CV (%)	4.1 [4.0, 5.2]	3.6 [3.2, 4.0]
Stride Length CV (%)	6.5 [5.8, 10.3]	4.4 [3.6, 5.2]
Step Length CV (%)	8.0 [6.1, 14.2]	5.7 [5.3, 6.7]
Gait Asymmetry (GA)	1.9 [0.5, 4.9]	3.9 [2.9, 4.5]
Phase Coordination Index (PCI) (%)	9.0 [7.3, 12.3]	7.8 [6.6, 8.1]

There were meaningful effect size changes among participants in quality of life subsections as per the PDQ-39 handbook (Table 4) [19]. These subsections included mobility, ADLs, emotional well-being, stigma, social support, cognitions, bodily discomfort, and the overall standard index score. Only one sub-dimension, communication, did not show meaningful change from baseline to end of training.

**Table 4:** Mean (n = 4) Effect Sizes in Quality of Life Domains Following Robot-Assisted Gait Training

	*Effect Size*
PDQ-39 SI*	-0.46
Mobility*	-0.20
ADLs*	-0.34
Emotional well-being*	-0.56
Stigma*	-0.49
Social Support*	-0.52
Cognitions*	-0.59
Communication	-0.06
Bodily Discomfort*	-0.21

*Denotes meaningful change in the PDQ-39 subsection score.^19^

### Clinician-Rated vFOG Outcome

**Figure 2:**
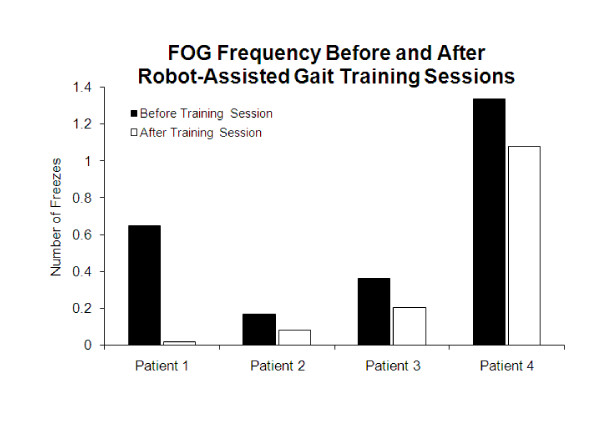
**Frequency vFOG scores (median of all scores, recorded before and after each training session)**.

**Figure 3:**
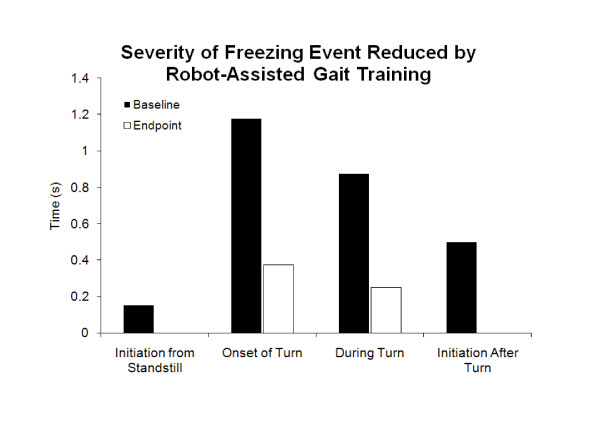
**Severity vFOG scores for all contexts (n = 4)**.

Median frequency vFOG scores improved 73.2% immediately following training sessions (Figure 2). Additionally, median frequency vFOG scores improved 62.5% from baseline to end of training. The severity of FOG was reduced in all walking contexts for all participants from baseline to end of training (Figure 3).

## Discussion

To our knowledge, this is the first study to examine the effects of robot-assisted BWSTT on FOG in individuals with PD+FOG. Our results showed that ten 30-minute sessions of robot-assisted treadmill training may reduce FOG frequency and severity, as well as abnormal gait variability, in a case series of four participants. Furthermore, we saw evidence for improved balance and decreased frequency of falls. The intervention also resulted in meaningful changes in seven of the eight quality of life dimensions, as well as in the overall PDQ-39 score. The vFOG scoring method demonstrated the possibility of evaluating FOG frequency and severity to assess changes after an intervention using videotaped sessions of five 10-meter walks including turns.

A previous study reported the directionally restricted effects of gait training on reducing FOG. Hong et al. (2008) used a rotating treadmill to improve FOG symptoms in two participants, but found that FOG decreased only in the trained direction [21]. In contrast, our study involved only continuous straight walking and no specific training for turns. We found decreased frequency of FOG during turn onset and after turning, as well as decreased severity of FOG for all aspects of turning (onset, during and after turning).

FOG-Q scores improved for severity of FOG episodes (questions 4-6), but not for frequency of FOG (question 3). The FOG-Q only has one question regarding FOG frequency compared to three questions on severity. Therefore, the FOG-Q may not be as sensitive to measure frequency of FOG. Total FOG-Q scores showed moderate improvement over the five week training protocol (2 points); this is less than what was reported by Frazzitta and colleagues (5.1 points), who also used a treadmill intervention to treat FOG [9]. The differences between the current study and Frazzitta et al. might be attributed to variations in both frequency and type of treadmill training paradigm. Frazzitta et al. incorporated a high intensity training protocol (20 min/day, every day for 4 weeks) into a multi-dimensional treadmill training paradigm augmented with auditory and visual cueing. In terms of gait changes, our results showed comparable improvements in gait velocity, despite the fewer number of sessions in our study (10 vs. 28 sessions). Furthermore, our study demonstrated a larger magnitude of change in gait velocity despite slower baseline gait velocities compared to the RESCUE trial examining cueing in individuals with PD+FOG [8].

Our results support the concept that individuals with PD+FOG exhibit abnormal gait patterns even in the absence of freezing episodes, which has been suggested previously [7]. Decreased stride length and increased step length variability have been attributed to increased FOG episodes [22-24]. We observed considerable improvements in stride length and step length CV after training, trending toward previously reported step length CV values for individuals with PD without FOG [22].

Furthermore, the results show improvement in overall gait coordination after treatment, as measured by PCI. PCI has been used to describe gait coordination in individuals with PD and PD+FOG [7,18,25]; however, change in PCI has not been examined as an outcome variable following intervention for individuals with PD+FOG. The participants in this study demonstrated improvements in overall PCI (9.0 to 7.8), approaching values previously reported for individuals with PD who do not experience FOG (6.95) [25]. While improvements were observed in all other measures pertaining to gait, this was not true for gait asymmetry (GA). This dichotomous change of improved coordination along with greater asymmetry may suggest that although gait patterns appear more asymmetrical, they are also more coordinated, consistent and rhythmic [18]. Similar changes in gait coordination versus GA are seen following levodopa treatment and results in a differential effect on improving PCI, but with no changes observed in GA [7].

Quality of life measures in the present study showed improvements in several domains investigated. Treadmill training has been shown to have beneficial effects on quality of life in individuals with PD only [26,27], while studies incorporating other methods of rehabilitation in individuals with PD+FOG have shown no changes in quality of life [8]. Results from the current study showed improvement in quality of life domains that might have been expected to benefit from treadmill training such as mobility and ADLs; however, additional beneficial effects were found on unexpected domains such as emotional well-being, cognition, and stigma.

This study was limited by the small number of participants and lack of a control group; there is the possibility that the changes observed may be due to a placebo effect or fluctuating responses to medication. Additionally, previous literature has suggested that treadmill training may be more beneficial than conventional physical therapy for improving gait in individuals with PD [28].

A potential limitation of prior FOG studies has been the reliance on using the self-reported FOG-Q. To address this limitation, our study included multiple methods to verify FOG. Our clinician-rated vFOG score demonstrated a reduction of FOG frequency and severity; however, there are several issues that should be addressed. Our initial intent was to develop a relatively simple walking task incorporating events similar to those in the FOG-Q and a previous study that assessed FOG through structured video assessment [17]; however, our 10-meter walking task did not provoke a high volume of freezing. Without a sufficient number of freezing episodes, it is difficult to document large changes due to treatment. The challenge of eliciting FOG episodes within the clinic, despite reports of FOG occurring at home, has been previously reported [5,29].

## Conclusions

These study results show that robot-assisted gait training is a promising therapy to reduce FOG events and improve gait parameters in participants with PD+FOG. The current study extends the knowledge of potential clinical therapeutic strategies and FOG outcomes used to treat and monitor gait abnormalities present in individuals with PD+FOG. Future studies should include clinician-rated measures assessing frequency and severity of FOG, as well as situations that elicit freezing, such as walking through narrow spaces and turning, since very few freezing events occur along straight pathways, as observed by this study and by Schaafsma et al. 2008 [17]. Furthermore, follow-up evaluations should be conducted to assess whether there are any long-term improvements from robot-assisted gait training.

## Competing interests

JHF has received funds for research, lectures or consulting from: Acadia Pharmaceuticals, Teva, Ingelheim-Boehringer, Glaxosmithkline, Cephalon, Valeant, EMD Serono, Pfizer, National Institute of Health, and Michael J Fox Foundation. All other authors declare that they have no competing interests.

## Authors' contributions

All authors read and approved the final manuscript. ACL was responsible for the conception, organization and execution of the project. He also assisted with developing the design and review and critique of the statistical analysis. Finally, he assisted in the preparation, review and critique of the manuscript. VCC helped to organize and execute the study. She also assisted with the statistical analysis and review of the manuscript. MAG assisted with the organization and execution of the study, as well as the statistical analysis, manuscript preparation and review. JHF was involved with the conception and execution of the study. He also assisted with statistical analysis and review of the manuscript. TSP assisted with the review and critique of the statistical analysis, as well as the preparation and review of the manuscript. DFB was involved with the execution of the study protocol and with the review of the manuscript.
